# Supplementary material for: Updated vaccine protects against SARS-CoV-2 variants including Omicron (B.1.1.529) and prevents transmission in hamsters
Source: Nat Commun. 2022 Nov 4;13:6644. doi: 10.1038/s41467-022-34439-7 (PMC9636174; doi:10.1038/s41467-022-34439-7)
Supplement: Supplementary file 1 — Supplementary Information [file 41467_2022_34439_MOESM1_ESM.pdf]

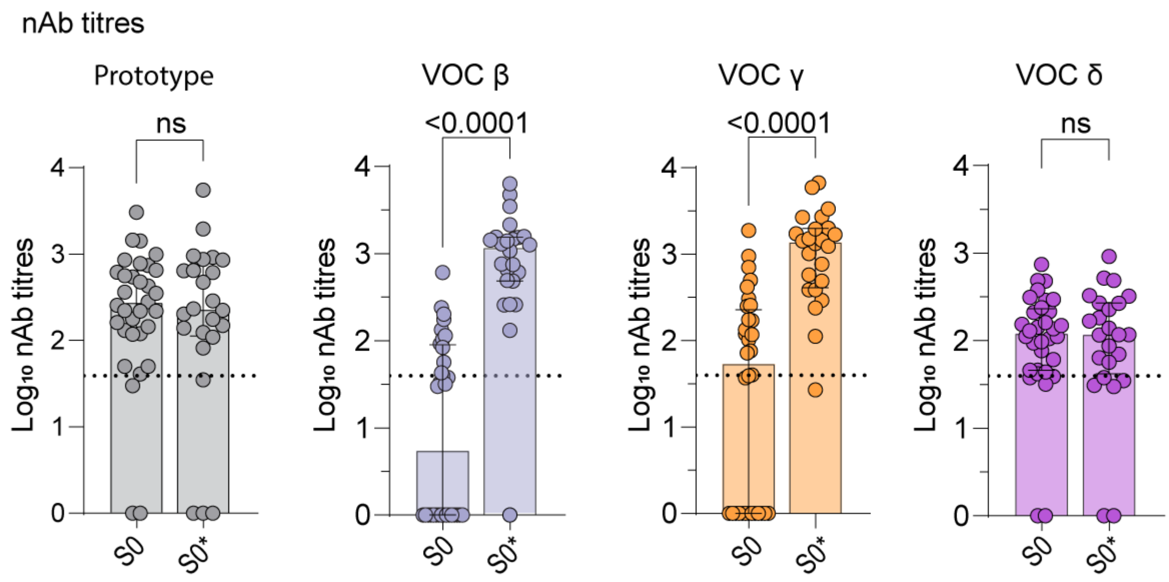

Figure S1 (related to main Figure 2A, D). Comparison of the immunogenicity of S0 and S0*. NAb titres against prototypic (grey circles), VOC Beta (blue circles), VOC Gamma (orange circles) and VOC Delta (purple circles) spike pseudotyped virus on day 21 after vaccination with prototype YF-S0 (*n*=32) and YF-S0* (*n*=24). Bar graphs denote median ± IQR. Dotted line represents the lower limit of quantification. Differences between groups were analyzed using non-parametric two-tailed Mann-Whitney.

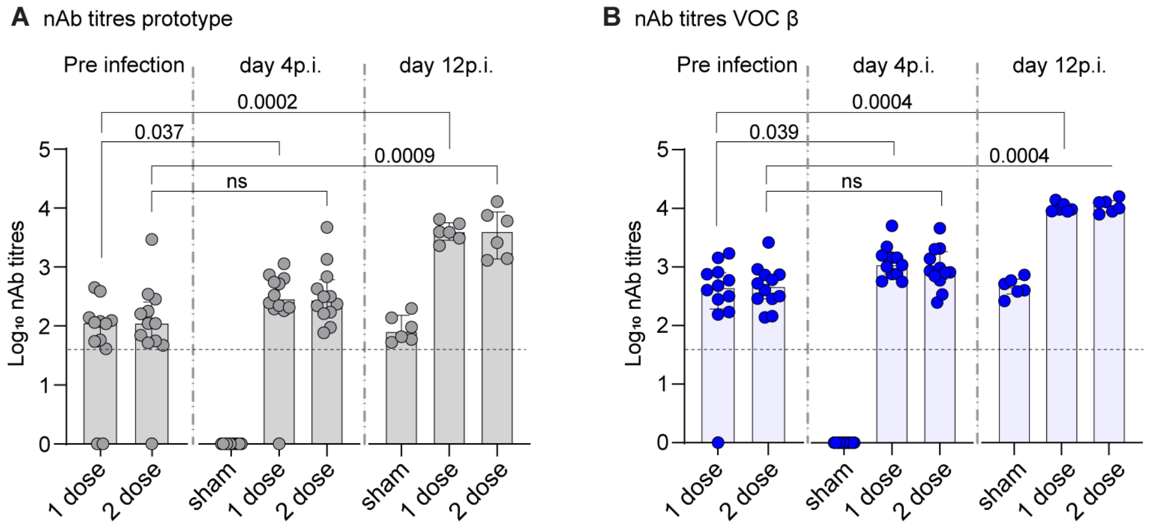

Figure S2 (related to main Figure 3). Comparison of nAb levels pre and post challenge. A, B, nAb titres against prototypic (grey circles) (**A**) and VOC Beta (blue circles) (**B**) spike pseudotyped virus on day 21 after vaccination with 1 (*n*=12) or 2 doses (*n*=12) of YF-S0* (pre infection) and day 4 (*n*=24 vaccinated and *n*=12 sham vaccinated) or day 12 (*n*=12 vaccinated and *n*=6 sham vaccinated) after infection. Bar graphs denote median ± IQR. Dashed line (horizontal) represents the lower limit of quantification. Pre infection nAb data have been reused from main Figure 3B. Differences between groups were analyzed using non-parametric Kruskal Wallis test uncorrected for ties.

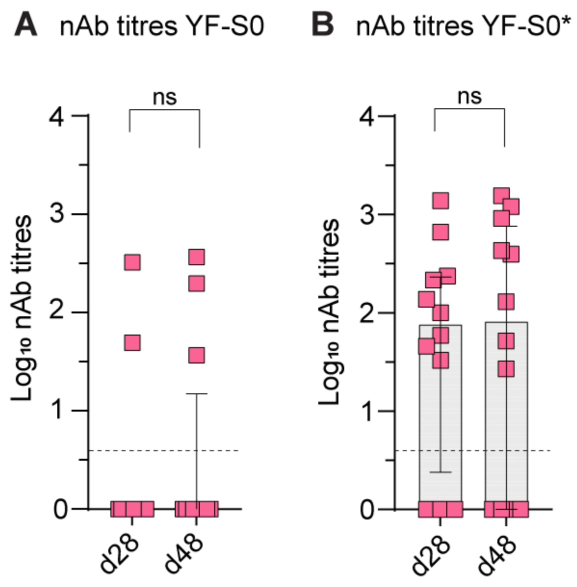

Figure S3 (related to main Figure 4). Increased potency of vaccine candidate S0* against VOC Omicron. A, B, nAb titres against omicron spike pseudotyped virus (red squares) on day 28 and day 48 after vaccination with two doses (day 0 and day 7) of prototype YF-S0 (*n*=12) (**A**) or YF-S0* (*n*=12) (**B**). Bar graphs denote median ± IQR. Dashed line represents the lower limit of quantification. Differences between groups were analyzed using non-parametric two-tailed Mann-Whitney.
