# Supplementary material for: Updated vaccine protects against SARS-CoV-2 variants including Omicron (B.1.1.529) and prevents transmission in hamsters
Source: Nat Commun. 2022 Nov 4;13:6644. doi: 10.1038/s41467-022-34439-7 (PMC9636174; doi:10.1038/s41467-022-34439-7)
Supplement: Supplementary file 3 — Reporting Summary [file 41467_2022_34439_MOESM3_ESM.pdf]

nature portfolio

Corresponding author(s): Kai Dallmeier

Last updated by author(s): Oct 18, 2022

Reporting Summary

Nature Portfolio wishes to improve the reproducibility of the work that we publish. This form provides structure for consistency and transparency in reporting. For further information on Nature Portfolio policies, see our Editorial Policies and the Editorial Policy Checklist.

Statistics

For all statistical analyses, confirm that the following items are present in the figure legend, table legend, main text, or Methods section.

n/a	Confirmed	
☐	☒	The exact sample size (*n*) for each experimental group/condition, given as a discrete number and unit of measurement
☐	☒	A statement on whether measurements were taken from distinct samples or whether the same sample was measured repeatedly
☐	☒	The statistical test(s) used AND whether they are one- or two-sided<br>*Only common tests should be described solely by name; describe more complex techniques in the Methods section.*
☒	☐	A description of all covariates tested
☒	☐	A description of any assumptions or corrections, such as tests of normality and adjustment for multiple comparisons
☐	☒	A full description of the statistical parameters including central tendency (e.g. means) or other basic estimates (e.g. regression coefficient) AND variation (e.g. standard deviation) or associated estimates of uncertainty (e.g. confidence intervals)
☐	☒	For null hypothesis testing, the test statistic (e.g. *F*, *t*, *r*) with confidence intervals, effect sizes, degrees of freedom and *P* value noted<br>*Give P values as exact values whenever suitable.*
☒	☐	For Bayesian analysis, information on the choice of priors and Markov chain Monte Carlo settings
☒	☐	For hierarchical and complex designs, identification of the appropriate level for tests and full reporting of outcomes
☒	☐	Estimates of effect sizes (e.g. Cohen's *d*, Pearson's *r*), indicating how they were calculated

Our web collection on statistics for biologists contains articles on many of the points above.

Software and code

Policy information about availability of computer code

Data collection	Thermo Fisher Scientific HCS Studio (v.6.6.0) software was used to quantify GFP expressing cells on a Cell Insight CX5/7 High Content Screening platform (Thermo Fischer Scientific) similar to our previous publications PMID: 33203860,PMID: 33260195. No other specific software was used for data collection.
Data analysis	All statistical analyses were performed using GraphPad Prism 9 software (GraphPad, San Diego, CA, USA). qRT-PCR data were analyzed by LightCycler® 96 SW 1.1 software.

For manuscripts utilizing custom algorithms or software that are central to the research but not yet described in published literature, software must be made available to editors and reviewers. We strongly encourage code deposition in a community repository (e.g. GitHub). See the Nature Portfolio guidelines for submitting code & software for further information.

Data

Policy information about availability of data

All manuscripts must include a data availability statement. This statement should provide the following information, where applicable:

- Accession codes, unique identifiers, or web links for publicly available datasets
- A description of any restrictions on data availability
- For clinical datasets or third party data, please ensure that the statement adheres to our policy

Source data are provided with this paper. All data supporting the findings in this study are also available from the corresponding author upon request.

Human research participants

Policy information about studies involving human research participants and Sex and Gender in Research.

Reporting on sex and gender	NA
Population characteristics	NA
Recruitment	NA
Ethics oversight	NA

Note that full information on the approval of the study protocol must also be provided in the manuscript.

Field-specific reporting

Please select the one below that is the best fit for your research. If you are not sure, read the appropriate sections before making your selection.

☒ Life sciences ☐ Behavioural & social sciences ☐ Ecological, evolutionary & environmental sciences

For a reference copy of the document with all sections, see nature.com/documents/nr-reporting-summary-flat.pdf

Life sciences study design

All studies must disclose on these points even when the disclosure is negative.

Sample size	Sample sizes were chosen as a balance between (i) the sample size to equal or exceed what has been established in other comparable studies (e.g. PMID: 33203860,PMID: 33260195; PMID: 32408338), (ii) capacity of the high-containment facilities, and (iii) limited availability of animals. Pivotal studies have been performed in independent biological repeats.
Data exclusions	No data were excluded
Replication	Principal data could be successfully replicated in duplicate experiments. In case of systematical variation between replicates, data points from each experiment received unique symbol shapes.
Randomization	No randomization methods were used and confounders were not controlled. Since all animals had the same age and roughly of the same weight; all are females, we believe there is no a real confounder to be considered in the study that may impact the outcome of the experiments.
Blinding	Animal caretakers following up animal husbandry (welfare and general health monitoring) were blinded to group allocation in the animal facility. Whereas technicians involved in experimental animals handling (immunization, infection, virus sampling and organ collection) needed for safety reasons to be unblinded regarding group allocation, downstream analysis of raw data (qPCR, titration and histology) was however done blindly without revealing of groups ID at the time of analysis.

Reporting for specific materials, systems and methods

We require information from authors about some types of materials, experimental systems and methods used in many studies. Here, indicate whether each material, system or method listed is relevant to your study. If you are not sure if a list item applies to your research, read the appropriate section before selecting a response.

Materials & experimental systems

n/a	Involved in the study	
☒	☐	Antibodies
☐	☒	Eukaryotic cell lines
☒	☐	Palaeontology and archaeology
☐	☒	Animals and other organisms
☒	☐	Clinical data
☐	☐	Dual use research of concern

Methods

n/a	Involved in the study	
☒	☐	ChIP-seq
☒	☐	Flow cytometry
☒	☐	MRI-based neuroimaging

Eukaryotic cell lines

Policy information about cell lines and Sex and Gender in Research

Cell line source(s)	VeroE6 (ATCC CRL-1586) and BHK-21J cells (Lindenbach & Rice JVI 1997 PMID: 9371625) were obtained from Peter Bredenbeek, LUMC, the Netherlands; A549-Dual hACE2-TMPRSS2 cells were from Invitrogen (Cat. No. a549d-cov2r); HEK-293T cells were sourced from ECACC (Cat. No. 12022001)
Authentication	No authentication of the cell lines was performed, however cell lines were sourced from ATCC or EDACC collections and/or specified before (Sanchez-Felipe et al. Nature 2021 PMID: 33260195).
Mycoplasma contamination	Cell lines were regularly tested to be negative for mycoplasma contamination (PlasmoTest Mycoplasma Detection Kit; Invivogen Cat. No. rep-pt1).
Commonly misidentified lines (See ICLAC register)	None of the commonly misidentified cell lines were used.

Animals and other research organisms

Policy information about studies involving animals; ARRIVE guidelines recommended for reporting animal research, and Sex and Gender in Research

Laboratory animals	6-8 weeks old Female Syrian hamsters (Mesocricetus auratus, strain RjHan:AURA) were purchased from Janvier Laboratories and kept per two in individually ventilated isolator cages (IsoCage N Bio-containment System, Tecniplast)
Wild animals	No wild animals were used in the study.
Reporting on sex	Sex was not considered in study design and was used only as per the availability of the animals
Field-collected samples	No field-collected samples were used in the study.
Ethics oversight	Housing conditions and experimental procedures were approved by the ethical committee of KU Leuven (license P55-2021), following institutional guidelines approved by the Federation of European Laboratory Animal Science Associations (FELASA).

Note that full information on the approval of the study protocol must also be provided in the manuscript.

Dual use research of concern

Policy information about dual use research of concern

Hazards

Could the accidental, deliberate or reckless misuse of agents or technologies generated in the work, or the application of information presented in the manuscript, pose a threat to:

No	Yes	
☒	☐	Public health
☒	☐	National security
☒	☐	Crops and/or livestock
☒	☐	Ecosystems
☒	☐	Any other significant area

Experiments of concern

Does the work involve any of these experiments of concern:

No	Yes	
☒	☐	Demonstrate how to render a vaccine ineffective
☒	☐	Confer resistance to therapeutically useful antibiotics or antiviral agents
☒	☐	Enhance the virulence of a pathogen or render a nonpathogen virulent
☒	☐	Increase transmissibility of a pathogen
☒	☐	Alter the host range of a pathogen
☒	☐	Enable evasion of diagnostic/detection modalities
☒	☐	Enable the weaponization of a biological agent or toxin
☒	☐	Any other potentially harmful combination of experiments and agents
